# Genetic recording of transient endothelial activation in distinct alveolar capillary cells during pulmonary fibrosis

**DOI:** 10.1038/s41421-024-00745-1

**Published:** 2024-12-03

**Authors:** Hongxin Li, Shaohua Zhang, Xiuzhen Huang, Zhenqian Zhang, Kuo Liu, Qing-Dong Wang, Alex F. Chen, Kathy O. Lui, Kun Sun, Bin Zhou

**Affiliations:** 1 CAS CEMCS-CUHK Joint Laboratories for Cardiovascular Sciences, New Cornerstone Science Laboratory, State Key Laboratory of Cell Biology, Shanghai Institute of Biochemistry and Cell Biology, Center for Excellence in Molecular Cell Science, Chinese Academy of Sciences, University of Chinese Academy of Sciences, Shanghai, China; 2 Key Laboratory of Systems Health Science of Zhejiang Province, School of Life Science, Hangzhou Institute for Advanced Study, University of Chinese Academy of Sciences, Hangzhou, Zhejiang China; 3 Bioscience Cardiovascular, Research and Early Development, Cardiovascular, Renal and Metabolism (CVRM), BioPharmaceuticals R&D, AstraZeneca, Gothenburg, Sweden; 4 Institute for Developmental and Regenerative Cardiovascular Medicine, Xinhua Hospital, School of Medicine, Shanghai Jiao Tong University, Shanghai, China; 5 CAS CEMCS-CUHK Joint Laboratories for Cardiovascular Sciences, Department of Chemical Pathology; and Li Ka Shing Institute of Health Sciences, Prince of Wales Hospital, The Chinese University of Hong Kong, Hong Kong, China; 6 Department of Pediatric Cardiology, Xinhua Hospital, Shanghai Jiao Tong University School of Medicine, Shanghai, China; 7 School of Life Science and Technology, ShanghaiTech University, Shanghai, China

**Keywords:** Biological techniques, Mechanisms of disease

Dear Editor,

Endothelial cells (ECs) and mesenchymal cells are both derived developmentally from the mesoderm but display different gene expressions and functions. During heart development, endothelial-to-mesenchymal transition (EndoMT) is functionally indispensable for forming cardiac valves^1,2^. In addition, EndoMT has also been reported in cardiac fibrosis induced by transverse aortic constriction (TAC), during which new fibroblasts are generated from adult ECs^3^. Indeed, inhibition of EndoMT significantly delays the severe progression of cardiac fibrosis after TAC^3^, highlighting EndoMT as a promising therapeutic target. However, some studies have demonstrated that the newly formed fibroblasts are originated from resident fibroblasts but not from EndoMT^1^, and even transient EndoMT was not likely to occur in cardiac ECs after TAC^2^. However, for pulmonary hypertension (PH), some evidence suggests artery endothelial cells acquire aSMA expression via EndoMT in vascular remodeling^4,5^. Due to the controversy of EndoMT in adult pathology, genetic tracing data with enhanced precision are needed to clarify its function. Here, we used a dual-recombinase-mediated genetic lineage tracing approach (Fig. 1a) to detect EndoMT in pulmonary fibrosis.

**Fig. 1:**
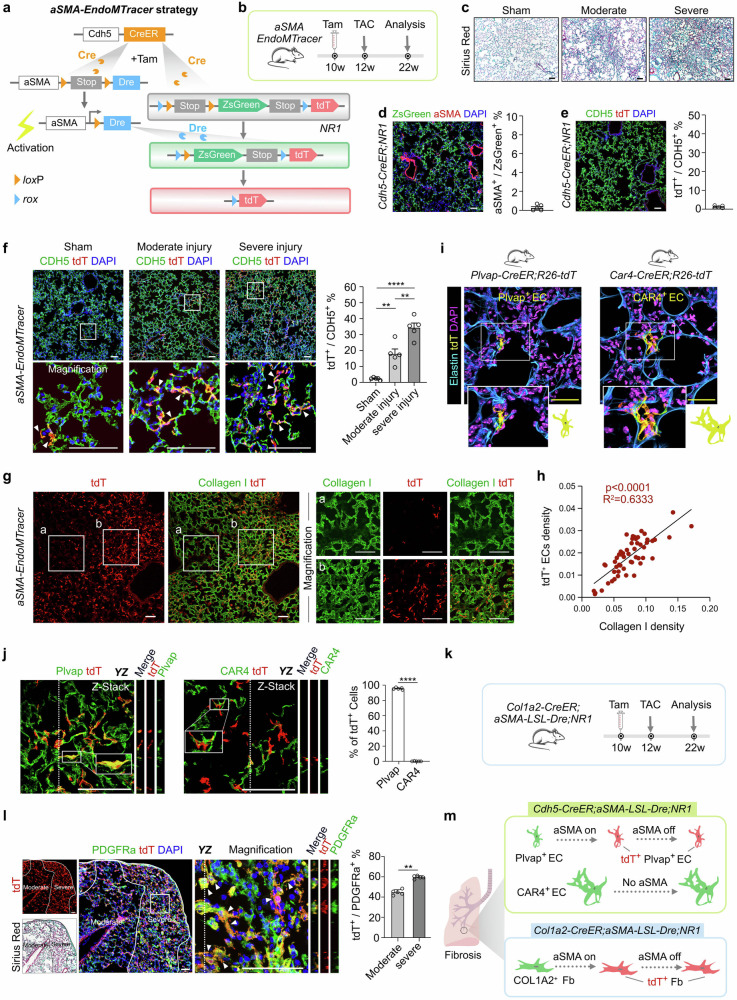
Transient aSMA activation in specific alveolar capillary ECs during pulmonary fibrosis. **a** Schematic diagrams illustrating the genetic approach employed to capture transient EndoMT. **b** Schematic showing the experimental design. **c** Sirius Red staining on the lung sections. **d**, **e** Immunostaining for ZsGreen and aSMA, Cdh5 and tdT on lung sections of *Cdh5-CreER;NR1* mice after TAC. Quantification histograms show the percentage of ZsGreen^+^ cells expressing aSMA, and CDH5^+^ cells expressing tdT. The data represent the mean ± SEM; *n* = 5. **f** Immunostaining for CDH5 and tdT on the lung sections of *aSMA-EndoMTracer* mice of Sham or TAC groups. White arrowheads indicate tdT^+^CDH5^+^ ECs. The right panel shows the quantification of the percentage of CDH5^+^ cells expressing tdT. The data represent the mean ± SEM; *n* = 5. **g** Immunostaining for tdT and Collagen I on the lung sections from *aSMA-EndoMTracer* mice after TAC. Region b exhibits a denser Collagen I and tdTomato distribution compared to region a. **h** Linear regression analysis of Collagen I density and tdT^+^ ECs density. **i** Immunostaining for tdT and Elastin on lung sections collected from *Plvap-CreER;R26-tdT* (left panel) and *Car4-CreER;R26-tdT* (right panel) mice. **j** Immunostaining for tdT and Plavp or CAR4 on lung sections after TAC. Right panel shows the quantification of the percentage of tdT^+^ cells expressing Plvap or CAR4. The data represent the mean ± SEM; *n* = 5, ****P < 0.0001. **k** Schematic showing the experimental design. **l** Immunostaining for PDGFRa and tdT on the lung sections. White arrowheads indicate tdT^+^PDGFRa^+^ cells. **m** Cartoon showing aSMA activation of Plvap^+^ but not CAR4^+^ ECs during pulmonary fibrosis (upper panel). COL1A2^+^ fibroblasts (Fb) express aSMA during lung fibrosis (lower panel). Scale bars, black and white, 100 μm; yellow, 50 μm.

TAC-induced chronic pressure overload causes vascular remodeling and degenerative pulmonary fibrosis, featured as right ventricular hypertrophy (Supplementary Fig. S1a, c) and leukocyte infiltration^6^. These changes resemble the histological remodeling observed in human PH secondary to left heart failure, which can be potentially fatal in the end stage. To record mesenchymal gene activation in ECs, we used a recently developed dual-recombinase genetic tracing strategy (Fig. 1a). In this system, *Cdh5-CreER* recombines *aSMA-LSL-Dre* after tamoxifen (Tam) induction, leading to the emergence of a new allele *aSMA-Dre* in ECs. Simultaneously, *Cdh5-CreER* recombines nested reporter 1 (*NR1*)^7^, genetically labeling CDH5^+^ ECs as ZsGreen. During pathological fibrosis in which *aSMA* gene is activated in ECs, even transiently, Dre would be expressed following *aSMA* promoter activation. The subsequent Dre-rox recombination would convert *NR1* from ZsGreen into tdTomato (tdT), allowing a permanent recording of transient *aSMA* gene activation in ECs. We treated adult *Cdh5-CreER;aSMA-LSL-Dre;NR1* (*aSMA-EndoMTracer*) mice with Tam, performed TAC in 2 weeks after Tam washout, and collected lungs for analysis at 10 weeks after TAC surgeries (Fig. 1b). By contrast, Sirius Red staining presents some regions in the lungs displayed a moderate to severe degree of fibrosis (Fig. 1c). We, therefore, examined whether any alveolar ECs could undergo mesenchymal gene activation after cardiac overload-induced pulmonary fibrosis. We first used the *Cdh5-CreER;NR1* mice that did not show any ZsGreen^+^SMA^+^ ECs after TAC, indicating that a single-recombinase-mediated genetic tracing strategy cannot seamlessly capture *aSMA* activation in alveolar ECs during fibrosis (Fig. 1d). We also did not detect tdT signal in alveolar ECs of *Cdh5-CreER;NR1* mice, excluding the potential cross-talk of Cre-rox recombination in our system (Fig. 1e).

Whole-mount fluorescence imaging revealed significant ZsGreen signals in the heart and lung of *aSMA-EndoMTracer* mouse in both sham and TAC groups. After TAC, tdT signals were observed in the lung but not in the heart (Supplementary Fig. S1b). We next performed immunostaining for CDH5 and tdT on lung sections. We observed sparse tdT^+^ ECs in the Sham group, indicating a minimal level of EC activation activity in the alveolar ECs during homeostasis (Fig. 1f). By contrast, we detected massive tdT signals in different lung lobes after TAC (Supplementary Fig. S1d), and there is a substantial increase in the proportion of tdT^+^ ECs in the TAC group: 18.07 ± 3.12% and 35.79 ± 3.98% from the moderate and severe fibrosis regions, respectively (Fig. 1f). Further Collagen I immunostaining revealed a significant positive linear correlation between tdT^+^ ECs density and Collagen I density (*P* < 0.0001, *R*^2^ = 0.6333) (Fig. 1g, h), suggesting transient endothelial cell activation events preferentially occurred in fibrotic areas. We also detected *aSMA* activation in ECs of larger diameter arteries and smaller diameter arterioles (Supplementary Fig. S2a, b). The TAC group exhibited a higher percentage of tdT^+^ signals (21.32 ± 2.53%) compared to the sham group (8.43 ± 1.07%). These tdT^+^ cells were identified as CDH5^+^, but not aSMA^+^ (Supplementary Fig. S2c–e).

We further attempted to identify the specific alveolar EC subsets that undergo *aSMA* activation during fibrosis. A recent study has shown that alveolar ECs consist of two distinctive yet intermingled cell types, Plvap^+^ general capillary (gCap), which is specialized for regulating vasomotor tone and functions as progenitors in vascular homeostasis and repair; and CAR4^+^ aerocyte, which is specialized for gaseous exchange^8,9^. We then used *Plvap-CreER* and *Car4-CreER* to label Plvap^+^ gCaps and CAR4^+^ aerocytes respectively, which showed distinct cell size and morphology as two distinct EC types in the alveoli (Fig. 1i)^10^. Immunostaining for tdT and Plvap or CAR4 on lung sections revealed that virtually all tdT^+^ ECs were Plvap^+^ but CAR4^–^ (Fig. 1j), suggesting that distinct alveolar ECs respond to lung injury.

Transition of fibroblasts to myofibroblasts is a normal process in wound healing, marked by *aSMA* expression^11^. We also generated *Col1a2-CreER;aSMA-LSL-Dre;NR1* mice to examine fibroblasts activation (Fig. 1k). Whole-mount fluorescence and sectional staining showed broad distribution of tdT^+^ cells in the injured heart and lung after TAC (Supplementary Fig. S3a–c). Consistent with previous research, immunostaining results showed that *aSMA* was activated in fibroblasts in cardiac fibrosis (Supplementary Fig. S3d)^2^. A substantial number of PDGFRa^+^ fibroblasts in the injured lung also exhibit tdT, and the percentage of PDGFRa^+^ fibroblasts expressing tdT were higher in the severe injured region than the moderate injured region (Fig. 1l). These data suggested that lung fibroblast expressed *aSMA* and regionally contributed to fibrosis during TAC-induced pulmonary fibrosis.

The development of pulmonary fibrosis is correlated with EC dysfunction. Capillary ECs have been implicated in the pathogenesis of PH, as their compromised barrier integrity disrupts homeostasis and impairs recruitment and activation of other cell types^12^. Here, we employed a dual-recombinase genetic lineage tracing system to demonstrate that lung alveolar capillary ECs undergo transient endothelial activation by exhibiting transient *aSMA* expression after cardiac TAC-induced pulmonary fibrosis, specifically occurring in Plvap^+^ ECs (Fig. 1m). Severer fibrosis was accompanied by more endothelial activation events, highlighting its potential contribution to chronic fibrosis, but further functional evidence such as inhibiting the signaling pathway of EndoMT is required to understand the regulatory mechanism. Moreover, it should be noted that transient endothelial activation does not negate the important role of fibroblasts in pulmonary fibrosis as massive *aSMA* activation was also observed in those activated fibroblasts. A subset of tdT^+^ fibroblasts (4.30 ± 0.66%) in the alveolar region were still positive for aSMA in *Col1a2-CreER;aSMA-LSL-Dre;NR1* mice, while minimal tdT^+^aSMA^+^ ECs (0.33 ± 0.14%) were detected in *aSMA-EndoMTracer* mice after TAC (Supplementary Fig. S3e–h). To investigate the significance of transient endothelial activation, we compared the gene expression pattern of the tdT^+^ and ZsGreen^+^ ECs by bulk RNA-sequencing analysis (Supplementary Fig. S4a, b). These tdT^+^ ECs may contribute to extracellular matrix remodeling by upregulating amino acid and lipid metabolism related genes^13^. Additionally, the upregulation of Rap1 signaling in tdT^+^ ECs may counteract vascular integrity maintenance and worsen the damage^14,15^ (Supplementary Fig. S4c–h).

Studies of ECs response during pulmonary fibrosis are instrumental to our understanding of the pathophysiological processes.

## Supplementary information

Genetic Recording of Transient Endothelial Activation in Distinct Alveolar Capillary Cells during Pulmonary Fibrosis
