# Supplementary material for: Genetic recording of transient endothelial activation in distinct alveolar capillary cells during pulmonary fibrosis
Source: Cell Discov. 2024 Dec 3;10:119. doi: 10.1038/s41421-024-00745-1 (PMC11612399; doi:10.1038/s41421-024-00745-1)
Supplement: Supplementary file 1 — Genetic Recording of Transient Endothelial Activation in Distinct Alveolar Capillary Cells during Pulmonary Fibrosis [file 41421_2024_745_MOESM1_ESM.pdf]

Supplementary Information for

Genetic Recording of Transient Endothelial Activation in Distinct Alveolar Capillary Cells during Pulmonary Fibrosis

Hongxin Li, Shaohua Zhang, Xiuzhen Huang, Zhenqian Zhang, Kuo Liu, Qing-Dong Wang, Alex F. Chen, Kathy O. Lui, Kun Sun*, Bin Zhou*

*Corresponding author: Bin Zhou and Kun Sun

Email: zhoubin@sibs.ac.cn

sunkun@xinhuamed.com.cn

This PDF file includes:

Materials and Methods

References

Supplementary figures

Materials and Methods

Mice

All mouse experiments in the study were strictly performed in accordance with the Institutional Animal Care and Use Committee (IACUC) guidelines of the Institute of Biochemistry and Cell Biology, Shanghai Institutes for Biological Sciences, Chinese Academy of Sciences. The mice were maintained on a C57BL6/129 mixed background, housed at specific-pathogen free (SPF) health status in individually ventilated cages under 12-12h light-dark cycles. The *Cdh5-CreER*, *Col1a2-CreER*, *aSMA-LSL-Dre*, *NR1*, *Plvap-CreER*, *Car4-CreER*, *R26-tdT* mouse line have been previously published[1-3]. *Cdh5-CreER* is transgenic, and others are knock-in mouse line via CRISPR-Cas9. In this study, a triple knock-in mouse line called *aSMA-EndoMTracer* (*Cdh5-CreER;aSMA-LSL-Dre;NR1*) was used to specifically capture the activation of the mesenchymal gene *aSMA* in endothelial cells. Similarly, another triple knock-in mouse line (*Col1a2-CreER;aSMA-LSL-Dre;NR1*) was utilized to specifically capture *aSMA* activation in fibroblasts. Additionally, *Plvap-CreER* and *Car4-CreER* mice were crossed with *R26-tdT* to accurately label distinct lung capillary populations.

Genomic PCR

Genomic DNA was extracted from mouse tails using a lysis buffer consisting of 100 mM Tris–HCl (PH 7.8), 5 mM EDTA, 0.2% SDS, 200 mM NaCl with 100 μg/mL protease K at 55°C overnight. After centrifugation, the supernatants were mixed with 95% ethanol to reach a final concentration of ethanol at 75%. This resulted in the conversion of soluble genomic DNA into flocculent precipitate. The DNA precipitate was then dissolved with ddH2O at 55℃. All mice were genotyped using genomic PCR as previously reported.

Whole-mount Fluorescence imaging

After euthanasia treatment, the mice were perfused with PBS to remove blood, followed by perfusion of 4% paraformaldehyde (PFA) through the trachea to fix the lungs. The collected lung and heart tissues were then washed with PBS and fixed in 4% PFA for 1 hour at 4℃. Subsequently, the tissues were thoroughly rinsed with PBS for several times. Tissues were placed on 1% agar gel and imaged using a Zeiss stereoscope (Axio Zoom.V16) for bright field and fluorescence imaging of whole-mount tissue samples.

Immunostaining and confocal imaging

After PFA fixing and PBS washing, the tissues were dehydrated overnight at 4°C in 30% sucrose solution. Next, the tissues were preembedded in optimum cutting tissue (O.C.T., Sakura) at 4°C for 1 hour. Subsequently, they were frozen at -20°C and stored at -80°C until ready to be sectioned. Cryosections of 10 μm thickness were then affixed onto adhesive slides. For immunostaining, tissue sections were air-dried at room temperature and washed O.C.T. with PBS. To block non-specific staining, the slices were incubated with the solution containing 0.2% Triton X-100, 5% normal donkey serum and DAPI (1:1,000) in PBS for 30 min at room temperature. The tissue slices were subsequently incubated with primary antibodies in PBST (0.2% Triton X-100 in PBS) overnight at 4°C. After that, the slices were washed with PBS for several times and incubated with secondary antibodies in PBST for 30 min at room temperature. For weak signals, tyramide signal amplification (TSA) was employed. Sections were washed in PBS and mounted with the mounting medium. Images were acquired by Zeiss LSM880 Ariyscan, Olympus FV4000 and analyzed utilizing ImageJ software.

The following primary antibodies were used in the study:

tdTomato (rabbit; 600-401-379; 1:1,000 dilution; Rockland), Plvap (rat; 553849; 1:500 dilution; BD pharmingen), CAR4 (goat; AF2414; 1:500 dilution; R&D), CDH5 (goat; AF1002; 1:100 dilution; R&D), ZsGreen (rabbit; 632474; 1:1000 dilution; Clontech), aSMA (mouse; c6198; 1:500 dilution; sigma), aSMA-fitc (mouse; F3777; 1:500 dilution; sigma), aPDGFRa (goat; AF1062; 1:200 dilution; R&D), Collagen I (rabbit; ab34710; 1:200 dilution; Abcam).

The following secondary antibodies were used in the study:

Alexa donkey anti-rabbit 555 (donkey; A31572; 1:1,000; Invitrogen), Alexa donkey anti-goat 647 (donkey; A21447; 1:1,000; Invitrogen), Immpress horse anti-rabbit (horse; MP-7401-50; 1:1; Vector lab), Alexa donkey anti-mouse 555 (donkey; A31570; 1:1,000; Invitrogen), Alexa donkey anti-mouse 488 (donkey; A21202; 1:1,000; Invitrogen), Alexa donkey anti-rat 647 (donkey; ab150155; 1:1,000; abcam), Alexa donkey anti-rabbit 647 (donkey; A31573; 1:1,000; Invitrogen).

Tamoxifen treatment

20 mg/mL Tamoxifen (Sigma-Aldrich, T5648) was dissolved in corn oil at room temperature. Tamoxifen treatment via oral gavage was administered at a dosage of 0.2 mg/g body weight to induce Cre-loxp recombination in adult. For sparse labeling, the adult mouse received Tamoxifen at a concentration of 2 mg/mL and a dose of 0.01mg/g body weight.

TAC model

Transverse aortic constriction (TAC) was performed in adult mouse as reported previously[4]. Male and female mouse were randomly selected in TAC group. The chest was opened without banding in the sham group.

Sirius Red Staining

Sirius red staining was performed to examine the degree of heart and pulmonary fibrosis. Tissue sections were fixed with 4% PFA for 15 min, and subsequently washed with PBS. The slides were immersed in Bouin solution (5% acetic acid, 9% formaldehyde, 0.9% picric acid) for 24 hours at room temperature. Following washing in running tap water, the slides were stained with 0.1% Fast green for 3-5 min. Next, the slides were washed with ddH2O and incubated with 1% Acetic acid for 1 min before staining with 0.1% Sirius Red for 1-3 min, followed by another round of ddH2O washing. Then the tissue sections were dehydrated successively in 95% ethanol for 2×3 min, 95% ethanol for 2×3 min, Xylene for 2×5 min. Finally, the slides were mounted with a resinous medium and preserved at room temperature.

FACS and bulk RNA sequencing

The digestive fluid of lung tissue consisted of 500 U/ml Collagenase I (Gibco, 17100-017), 2mg/ml Elastase (worthington, LS002279), 0.5mg/ml DNase I (worthington, LS002139), and RPMI-1640 medium (ThermoFisher, 11875093). Each mouse required a volume of 5ml digestive fluid. After euthanizing the mice, the digestive fluid was injected into the lungs via the trachea. The lungs were then excised and digested at 37°C for 30 minutes. Digestion was terminated by adding fetal bovine serum (FBS, ThermoFisher, 10099141c) to achieve a final concentration of 20%. The suspended cells were lysed using 1ml Red blood cell buffer (eBioscience, 00-4333-57) and the reaction was halted with PBS containing 1% BSA after an incubation period of 5 minutes. The antibodies employed for cell staining included PECAM-1(CD31) APC (eBioscience, 17-0311-82, 1:40 dilution), CD45 PE-Cy7 (eBioscience, 25-0451-82, 1:400 dilution). Staining took place at a temperature of 4°C for a duration of 30 minutes. Following staining, the cells were resuspended in PBS containing 1% FBS and subsequently sorted using BD FACSAriaFusion. The RNA sequencing was performed by BGI and the RNA-sequencing data generated in this study is deposited in NCBI GEO: GSE277471.

Statistics analysis

Prism software (GraphPad, v8.2.1) was used for statistical analysis. The statistics data were presented as mean ± SEM of biological replicates. For statistical analysis, unpaired Student's *t*-test was used to compare each two groups of mice The following P values were used: ****$P < 0.0001$, **$P < 0.01$.

References

1 Zhang, Z. & Zhou, B. Generation of Plvap-CreER and Car4-CreER for genetic targeting of distinct lung capillary populations. *J Genet Genomics* 49, 1093-1100 (2022).

2 Zhang, S. et al. Seamless Genetic Recording of Transiently Activated Mesenchymal Gene Expression in Endothelial Cells During Cardiac Fibrosis. *Circulation* 144, 2004-2020 (2021).

3 He, L. et al. Enhancing the precision of genetic lineage tracing using dual recombinases. *Nat Med* 23, 1488-1498 (2017).

4 Tang, J. et al. Genetic Fate Mapping Defines the Vascular Potential of Endocardial Cells in the Adult Heart. *Circ Res* 122, 984-993 (2018).

Supplementary Figures

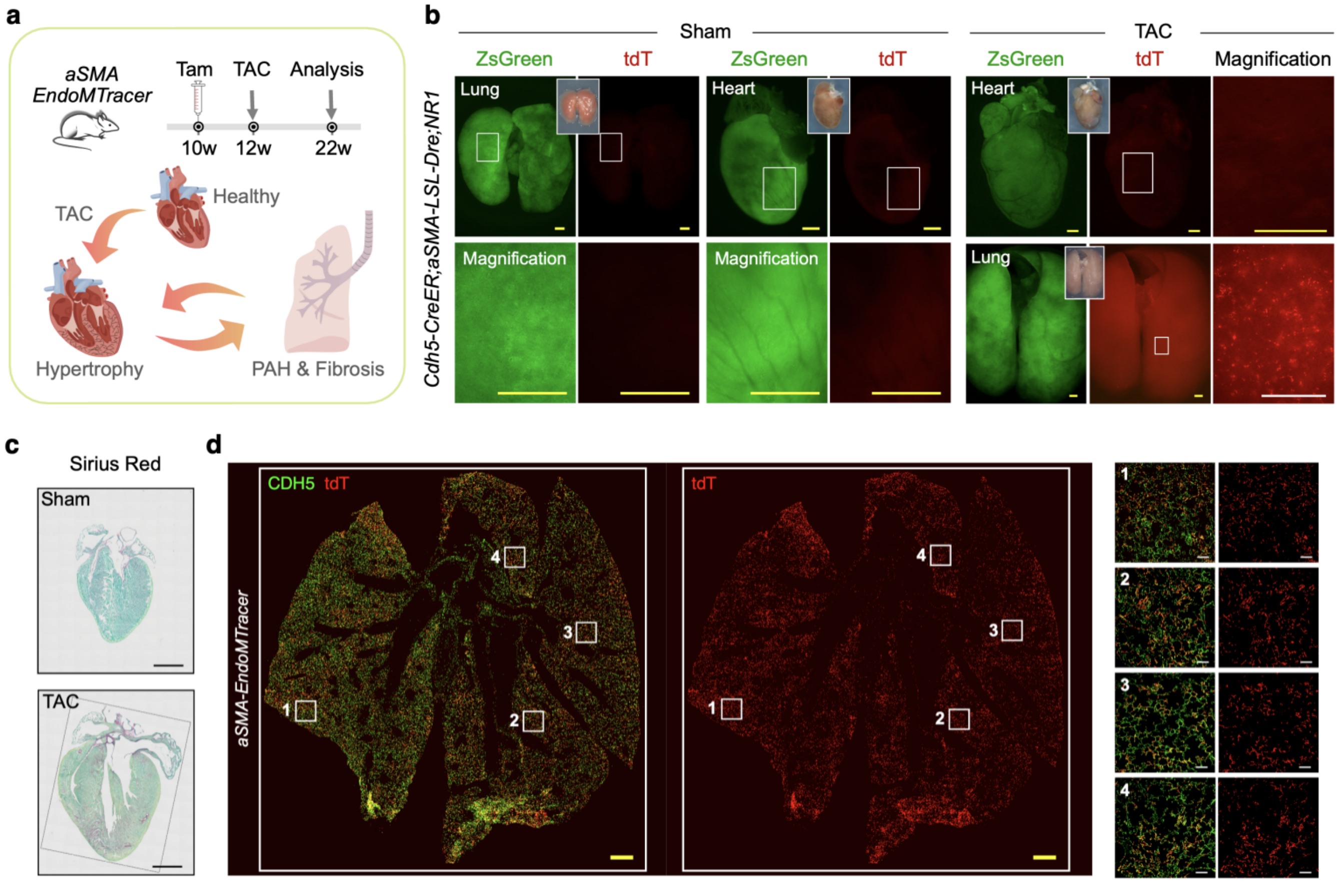

Supplementary Fig. S1 | Whole-mount tissue signature of *aSMA-EndoMTracer* mice after TAC **a.** Schematic showing the experimental design and the process of TAC inducing lung fibrosis. **b.** Whole-mount fluorescence of heart and lung tissues from *aSMA-EndoMTracer* mouse of sham and TAC group. Scale bars, yellow, 1mm; white, 500μm. **c.** Sirius Red staining on the heart sections. Scale bars, 2mm. **d.** Whole-mount sectional immunostaining for CDH5 and tdT on the lung section from *aSMA-EndoMTracer* mice post TAC. Scale bars, yellow, 1mm; white, 100μm.

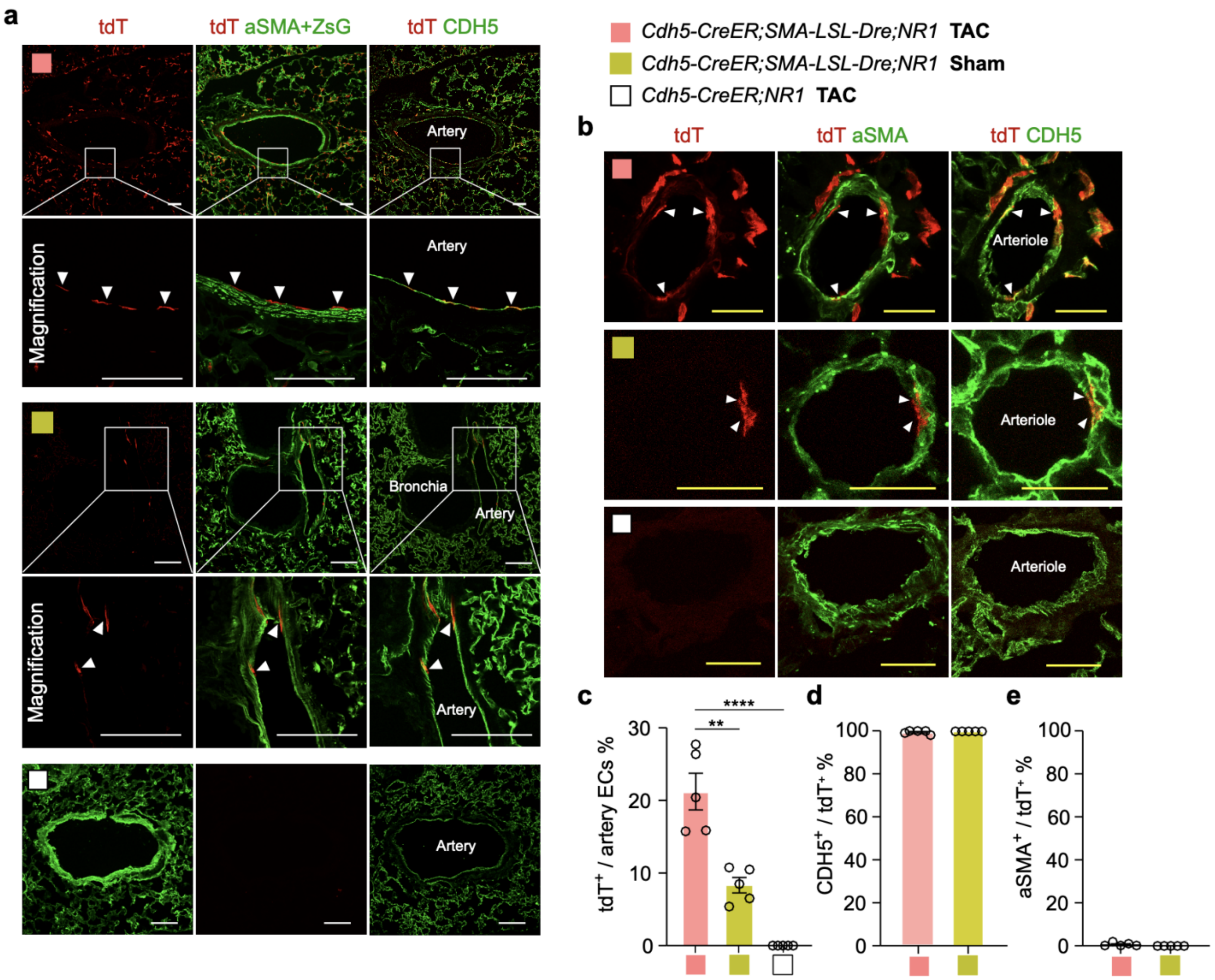

Supplementary Fig. S2 | Revealing tdT$^+$ ECs of pulmonary arteries and arterioles **a-b.** Immunostaining for tdT, sSMA, ZsGreen and CDH5 on the lung sections from *Cdh5-CreER;SMA-LSL-Dre;NR1*(*aSMA-EndoMTracer*) mouse of sham and TAC group and *Cdh5-CreER;NR1* mouse post TAC. Panel ***a*** showing the arteries with a larger diameter, white scale bars, 100µm. Panel ***b*** showing smaller diameter arterioles, yellow scale bars, 25µm. **c.** Quantification of the percentage of arterial ECs (including arteriolar ECs) expressing tdT (mean ± SEM, n=5). **P value<0.01, ****P value<0.0001. **d.** Quantification of the tdT$^+$ ECs expressing CDH5 (mean ± SEM, n=5). **e.** Quantification of the tdT$^+$ ECs expressing aSMA (mean ± SEM, n=5).

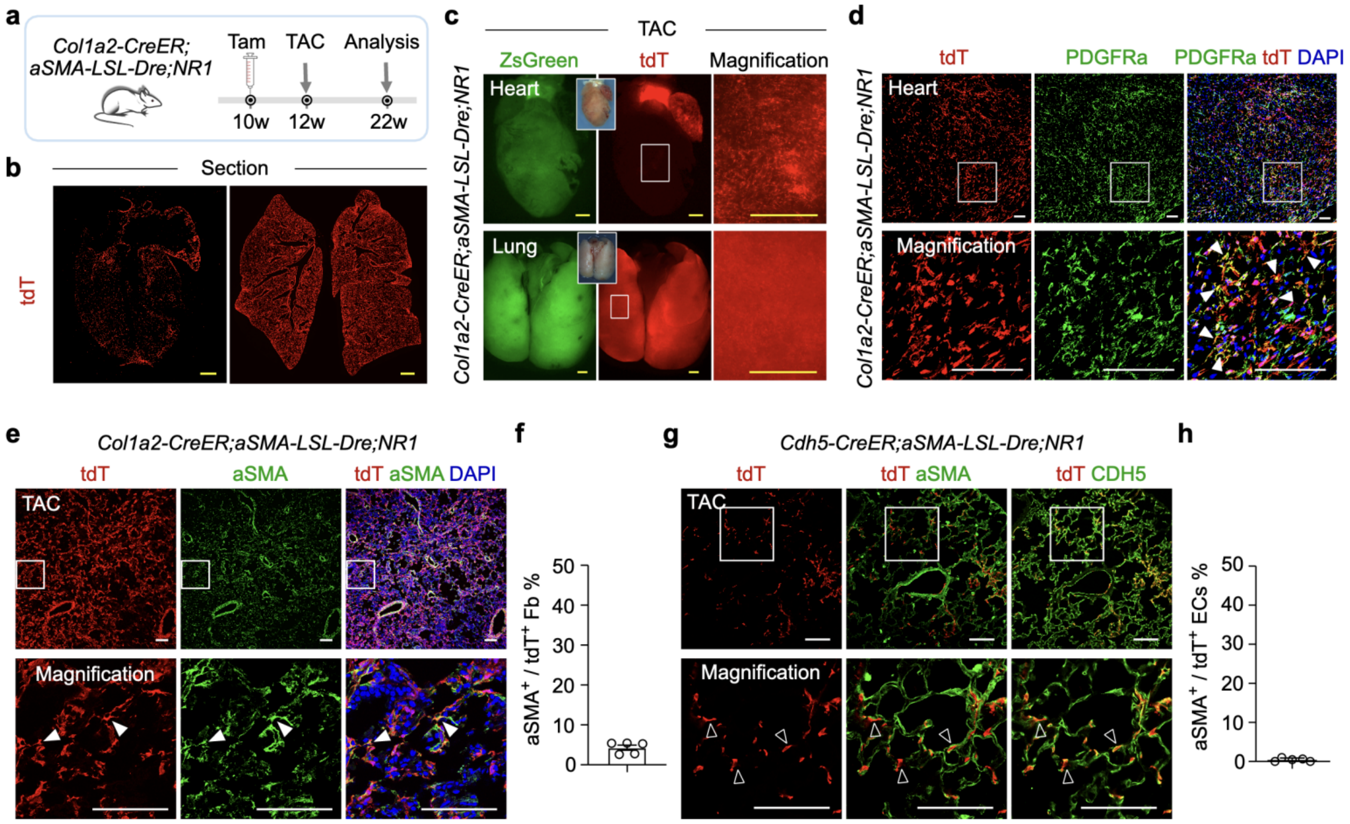

Supplementary Fig. S3 | Recording *aSMA* activation in fibroblasts during TAC-induced cardiac and pulmonary fibrosis. **a.** Schematic showing the experimental design. **b-c.** Whole-mount fluorescence of the heart and lung tissues of *Col1a2-CreER;SMA-LSL-Dre;NR1* mice after TAC. Scale bars, 1mm. **d.** Immunostaining for PDGFRa and tdT on the heart after TAC. Arrowheads indicate tdT^+ $PDGFRa^+$ fibroblasts. Scale bars, 100μm. **e-f.** Immunostaining for tdT and aSMA on the lung sections from *Col1a2-CreER;SMA-LSL-Dre;NR1* mice post TAC, quantification showing the percentage of tdT^+ fibroblasts (in the alveolar region) expressing aSMA. Arrowheads indicate tdT^+ $aSMA^+$ fibroblasts. Scale bars, 100μm. **g-h.** Immunostaining for tdT and aSMA on the lung sections from *Cdh5-CreER;SMA-LSL-Dre;NR1*(*aSMA-EndoMTracer*) mice post TAC 10 weeks, quantification showing the percentage of tdT^+ ECs expressing aSMA. Arrowheads indicate tdT^+ $aSMA^-$ $CDH5^+$ ECs. Scale bars, 100μm.

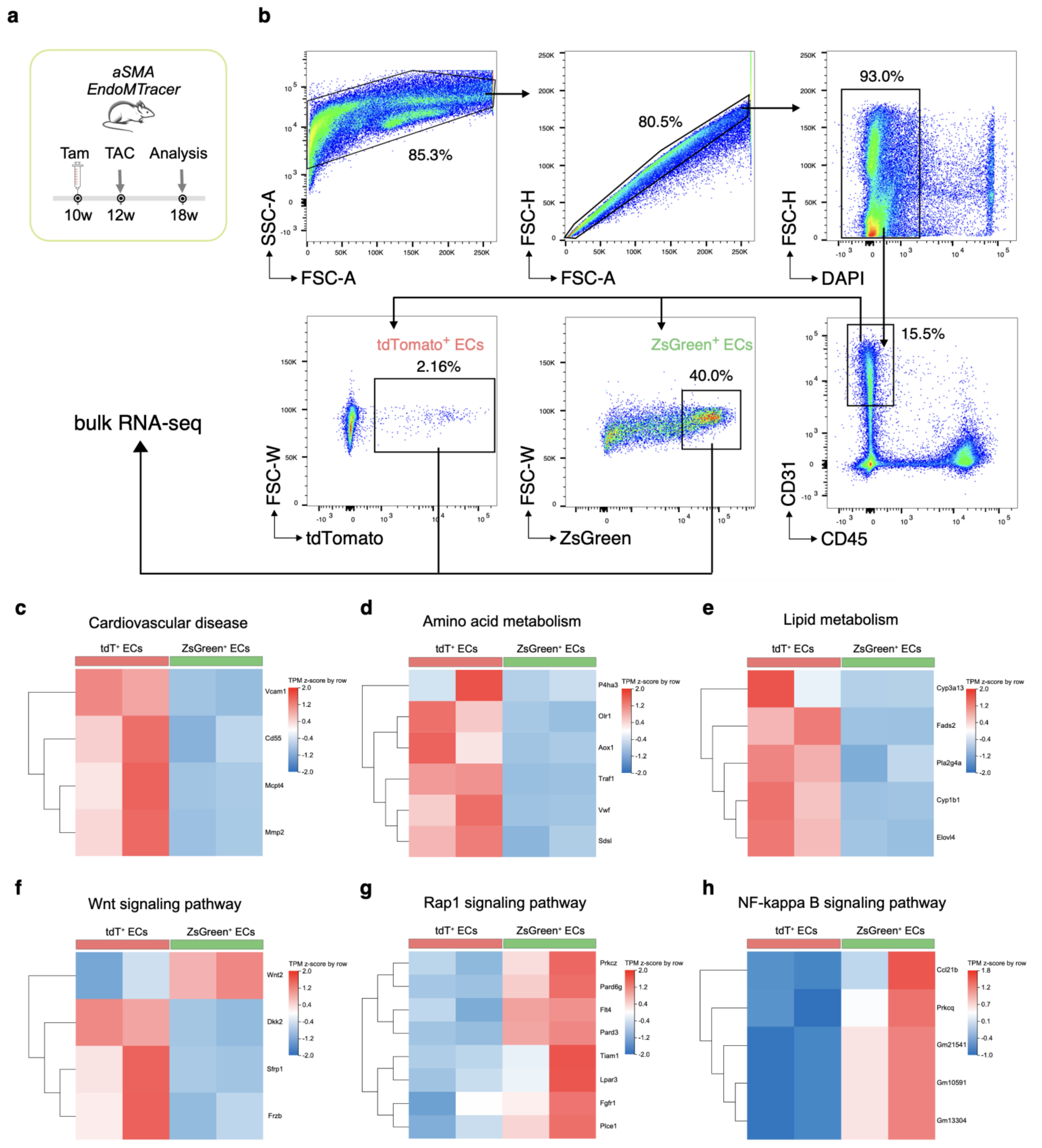

Supplementary Fig. S4 | FACS and bulk RNA-seq of pulmonary endothelial cells **a.** Schematic showing the experimental strategy. **b.** Flow cytometry gating strategy for sorting pulmonary endothelial cells. **c-h.** Heatmaps of top differentially expressed genes according to KEGG pathway term.
